# The Yeast PUF Protein Puf5 Has Pop2-Independent Roles in Response to DNA Replication Stress

**DOI:** 10.1371/journal.pone.0010651

**Published:** 2010-05-14

**Authors:** Ana Traven, Tricia L. Lo, Trevor Lithgow, Jörg Heierhorst

**Affiliations:** 1 Department of Biochemistry and Molecular Biology, Monash University, Melbourne, Victoria, Australia; 2 Molecular Genetics Unit, St. Vincent's Institute of Medical Research, Melbourne, Victoria, Australia; University College Dublin, Ireland

## Abstract

PUFs are RNA binding proteins that promote mRNA deadenylation and decay and inhibit translation. Yeast Puf5 is the prototype for studying PUF-dependent gene repression. Puf5 binds to the Pop2 subunit of the Ccr4-Pop2-NOT mRNA deadenylase, recruiting the deadenylase and associated translational repressors to mRNAs. Here we used yeast genetics to show that Puf5 has additional roles *in vivo* that do not require Pop2. Deletion of *PUF5* caused increased sensitivity to DNA replication stress in cells lacking Pop2, as well as in cells mutated for two activities recruited to mRNAs by the Puf5-Pop2 interaction, the deadenylase Ccr4 and the translational repressor Dhh1. A functional Puf5 RNA binding domain was required, and Puf5 cytoplasmic localisation was sufficient for resistance to replication stress, indicating posttranscriptional gene expression control is involved. In contrast to DNA replication stress, in response to the cell wall integrity pathway activator caffeine, *PUF5* and *POP2* acted in the same genetic pathway, indicating that functions of Puf5 in the caffeine response are mediated by Pop2-dependent gene repression. Our results support a model in which Puf5 uses multiple, Pop2-dependent and Pop2-independent mechanisms to control mRNA expression. The Pop2-independent roles for Puf5 could involve spatial control of gene expression, a proposition supported by our data indicating that the active form of Puf5 is localised to cytoplasmic foci.

## Introduction

PUF proteins are a family of RNA binding proteins conserved in eukaryotes, with roles in cell growth, division, differentiation and development [1], [2]. PUFs bind to 3′ untranslated regions of mRNAs and repress their expression. In many cases, they promote shortening of poly(A) tails (deadenylation), with the length of tail controlling both mRNA stability and translation [1]–[4]. Translational repression by PUFs that is independent of deadenylation has also been observed [5]–[8].

Studies with yeast Puf5 elucidated the mechanism of PUF-dependent repression. Puf5 binds directly to the Pop2 subunit of the Ccr4-Pop2-NOT mRNA deadenylase, thereby recruiting the complex to targeted mRNAs [6]. A functional interaction between Puf5 and Pop2 is also supported by earlier evidence that Puf5 can act as a multi-copy suppressor of certain phenotypes resulting from a *pop2* mutation [9]. The Pop2-Puf5 interaction recruits to mRNAs at least two repressive activities: (i) the deadenylase activity of Ccr4 and (ii) a deadenylase-independent function [6]–[8]. A candidate effector for this latter function is the translational repressor and activator of mRNA decapping Dhh1 [10], which is physically associated with Ccr4-Pop2-NOT [11] and is recruited to mRNAs by the Puf5-Pop2 interaction [6].

Puf5 dependent repression of the *HO* mRNA is abolished in the absence of Pop2, indicating that Pop2-mediated mechanisms are essential [6]–[8]. Moreover, the PUF-Pop2 interaction is conserved in *C. elegans*, *Drosophila* and humans [6], [12], [13], suggesting this might be a universal mode of PUF protein action [1].

Other yeast PUFs (Puf1, Puf3 and Puf4) also cause deadenylation and decay of their target mRNAs [8], [14], [15]. For Puf4, a functionally relevant interaction with Pop2 has been demonstrated [8].

Although many interacting mRNAs have been identified for each of the five yeast PUF proteins [16], relatively few direct links between PUF-mediated repression and *puf* mutant phenotypes have been reported [17]–[20]. Moreover, it is still poorly understood how relevant Pop2-dependent repression is for the cellular roles of the PUFs.

One way to address this question is by a genetic approach. Do mutations in the Ccr4-Pop2 deadenylase and the PUFs lead to analogous phenotypes, and if so do the genes act in the same genetic pathway? Both Pop2 and Puf5 are required for resistance to the DNA replication inhibitor hydroxyurea (HU) [21]–[23], and both *puf5* and *pop2* mutants have phenotypes consistent with defects in the cell wall [9], [19], [24]. We used these common phenotypes to dissect the functional relationship between Puf5 and Pop2.

## Results

### PUF5 acts independently of POP2 to promote resistance to DNA replication stress

**Figure 1:**
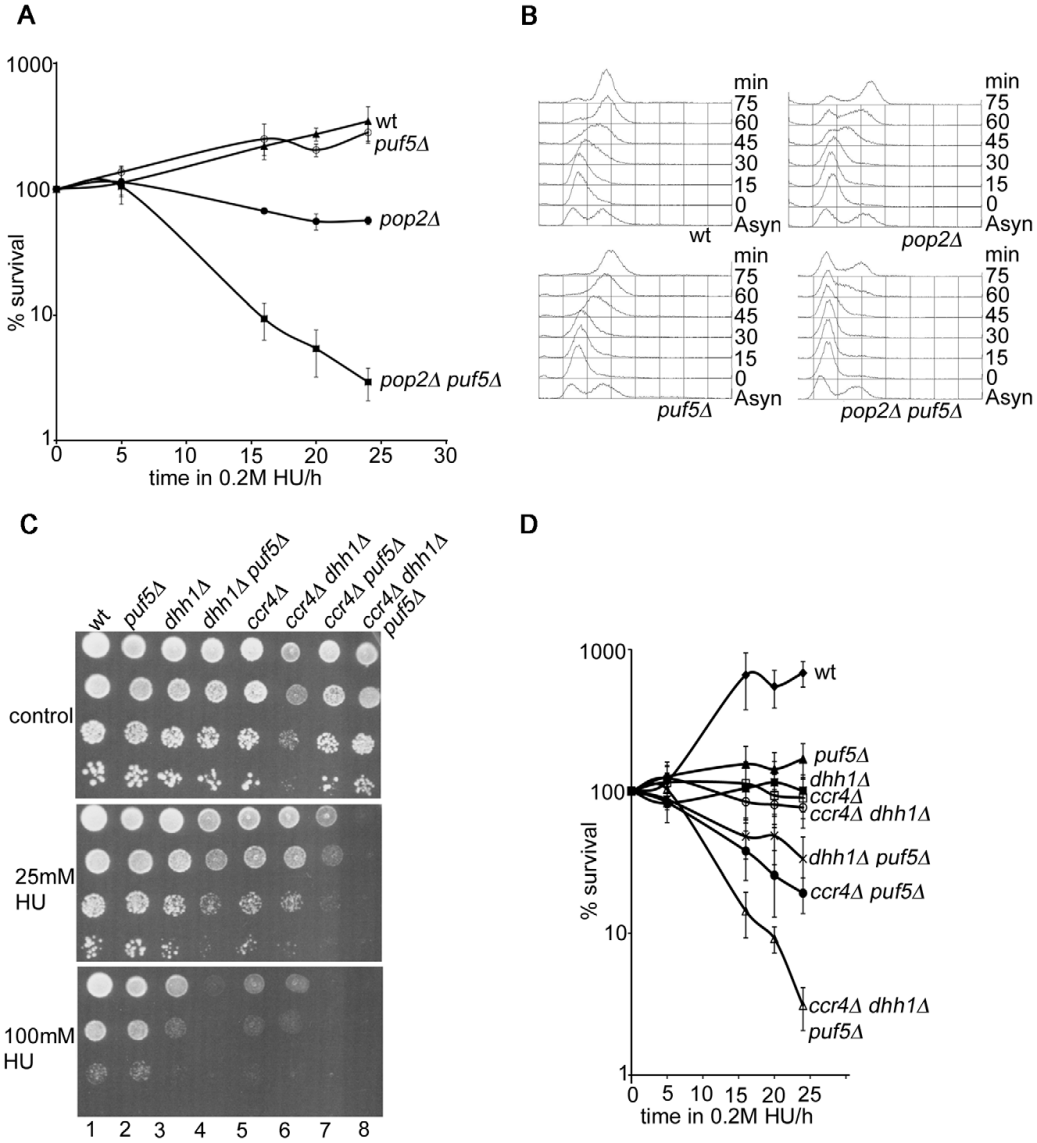
Puf5 has Pop2-independent roles in response to HU. A) Time course experiments were performed in 0.2 M HU. Shown are averages from three independent cultures and the standard deviation. B) Cells were processed for FACS analysis as described in Materials and Methods. C) Cells of the indicated strains were dropped on plates with or without HU and photographed after 3 days at 30°C. D) Time course experiments were performed as in A). Shown are averages of at least six independent colonies for each strain. The error bar is the standard deviation.

Pop2 is essential for Puf5-mediated gene repression [6]. This predicts that Puf5 functions will be inactivated in a *pop2Δ* strain, and therefore deletion of *PUF5* in a *pop2Δ* background should not cause further increase in the severity of phenotypes. However, in the case of sensitivity to HU, we found the opposite: deletion of *PUF5* caused increased sensitivity to HU in the *pop2Δ* mutant. Only 3% of double mutants survived after 24 h in 0.2 M HU, compared to 56% of *pop2Δ* cells (Figure 1A). Synthetic HU sensitivity was observed in independently constructed *pop2Δ puf5Δ* mutants and was complemented by plasmid-borne *PUF5* (Figure S1). FACS analysis showed that *pop2Δ puf5Δ* cells were delayed in cell cycle progression after S-phase arrest with HU (0 time point in Figure 1B) and even after 75 min showed a substantial S-phase peak (Figure 1B; re-start of DNA replication and moving towards G2 DNA content was obvious after 30 minutes in the wild type and *puf5Δ*, whereas the *pop2Δ* mutant was 15 minutes delayed). Delayed recovery of *pop2Δ puf5Δ* compared to the *pop2Δ* is consistent with synthetic HU sensitivity.

To corroborate the result that Puf5 has Pop2-independent roles in response to HU, we inactivated Puf5 in a strain lacking the two activities recruited to mRNAs by Pop2-Puf5, the deadenylase Ccr4 and the translational repressor Dhh1. The triple *ccr4Δ dhh1Δ puf5Δ* mutant exhibited severe synthetic hypersensitivity to HU, on plates and in time course survival assays (Figure 1C and D), confirming that Puf5 has roles in HU that are independent of Pop2-mediated gene repression.

**Figure 2:**
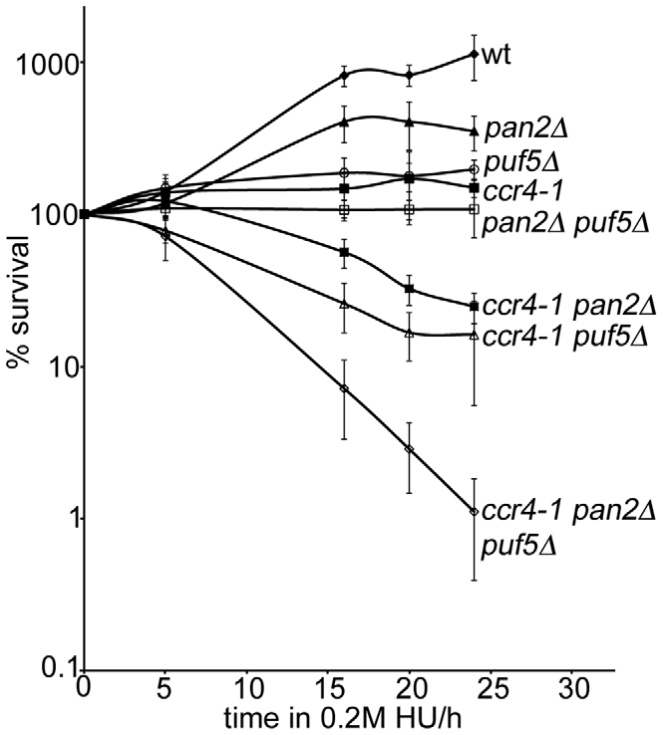
Puf5 has roles in response to HU that are independent of deadenylation. Survival in 0.2 M HU of the indicated strains was assayed over a 24 h time course. Shown are averages of six independent colonies and the standard deviation.

Because PUFs cause mRNA deadenylation [1], [2], we next tested if Puf5 acted via a different deadenylase. The only other yeast deadenylase Pan2-Pan3 is also required for survival in HU (25). To test if Puf5 acted via Pan, we deleted *PUF5* in a *ccr4-1 pan2Δ* double mutant *(ccr4-1* being a catalytically inactive allele of *CCR4*) [26], in which deadenylation is fully inactivated [27]. The triple mutant displayed a dramatic increase in HU sensitivity, dying 16 fold more than the sickest double mutant *ccr4-1 puf5Δ* (Figure 2). Therefore, the role of Puf5 in survival after HU treatment is deadenylation-independent.

**Figure 3:**
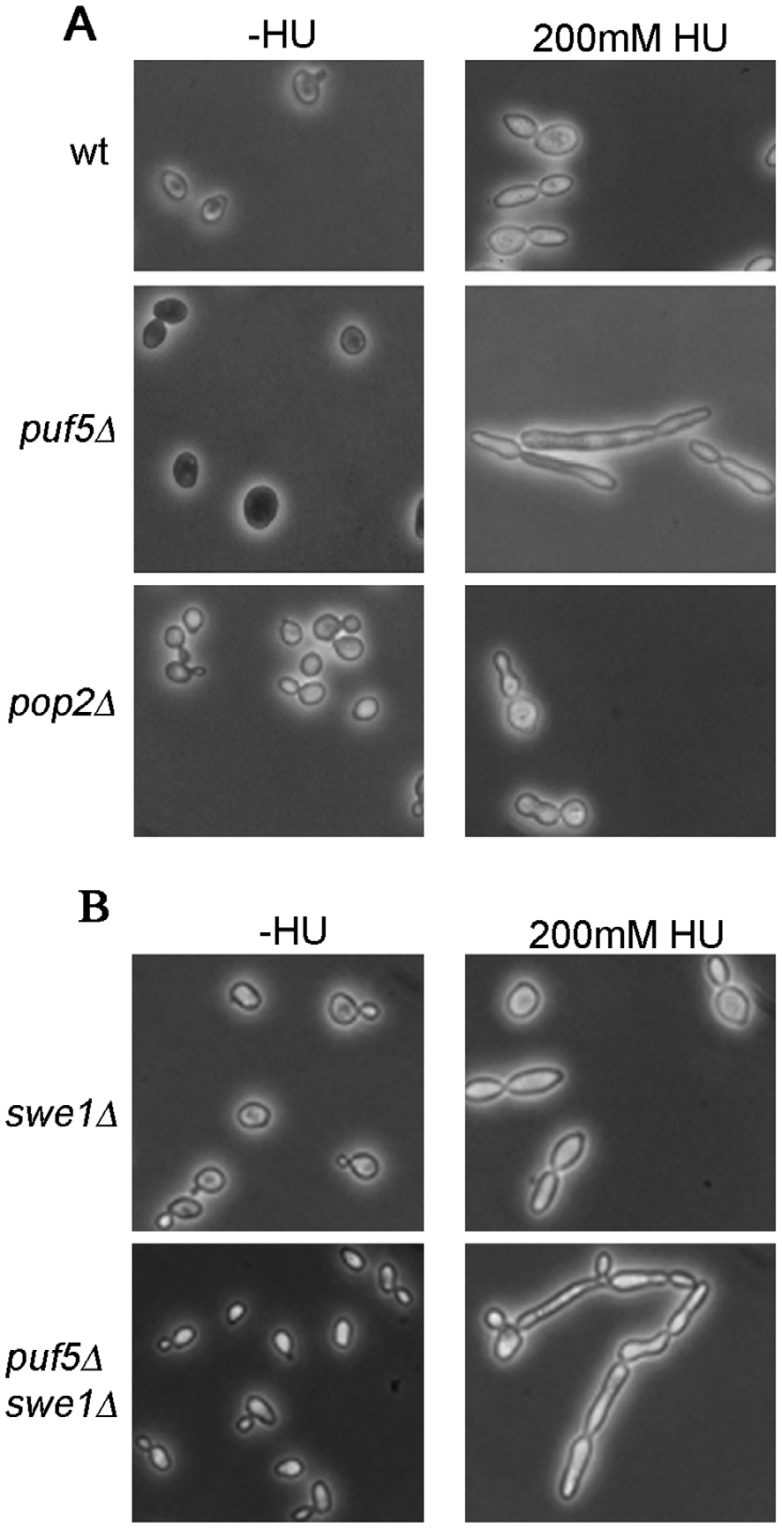
*puf5* mutants display a Swe1-independent elongated cell morphology in response to HU. Cells of the indicated strains were grown in the presence or absence of 0.2 M HU for 16 h, fixed in 70% ethanol, rehydrated in PBS and viewed.

HU causes pseudohyphal differentiation [28] and we observed that the *puf5Δ* mutant displayed a hyper-elongated cell morphology in response to HU treatment (Figure 3A). This result is consistent with a previous report on the role of Puf5 as a negative regulator of nitrogen starvation-induced filamentous differentiation [20]. We previously reported that a *pop2Δ* mutant also displays an elongated morphology in response to HU treatment [29]. However, the morphology of the two mutants is very different: *pop2Δ* cells have a “bud-chain” morphology after HU treatment (Figure 3A) and [29], which is due to the activation of the morphogenesis checkpoint kinase Swe1, and can be suppressed by deletion of the *SWE1* gene [29]. In contrast, cultures from *puf5Δ* mutants have elongated cells, which do not display “bud-chains” (Figure 3A); moreover, the hyper-elongation phenotype of *puf5Δ* cannot be suppressed by deletion of *SWE1* (Figure 3B). The different morphology of the *puf5Δ* and *pop2Δ* mutants in response to HU, and the difference in genetic requirements for the morphogenesis phenotypes, are consistent with Pop2-independent roles for Puf5 in response to HU.

### The roles of Puf5 in response to HU are separable from its roles in response to the cell wall integrity pathway activator caffeine

In addition to HU sensitivity, both Puf5 and Pop2 are required for resistance to agents that perturb cell walls [9], [19], [24], such as the cell wall integrity pathway activator caffeine [9]. Importantly, links between DNA damage responses and cell wall integrity have been reported [30]–[32]. For example, the cell wall integrity pathway kinases Pkc1 and Slt2 are required for resistance to HU-induced DNA replication stress [30].

**Figure 4:**
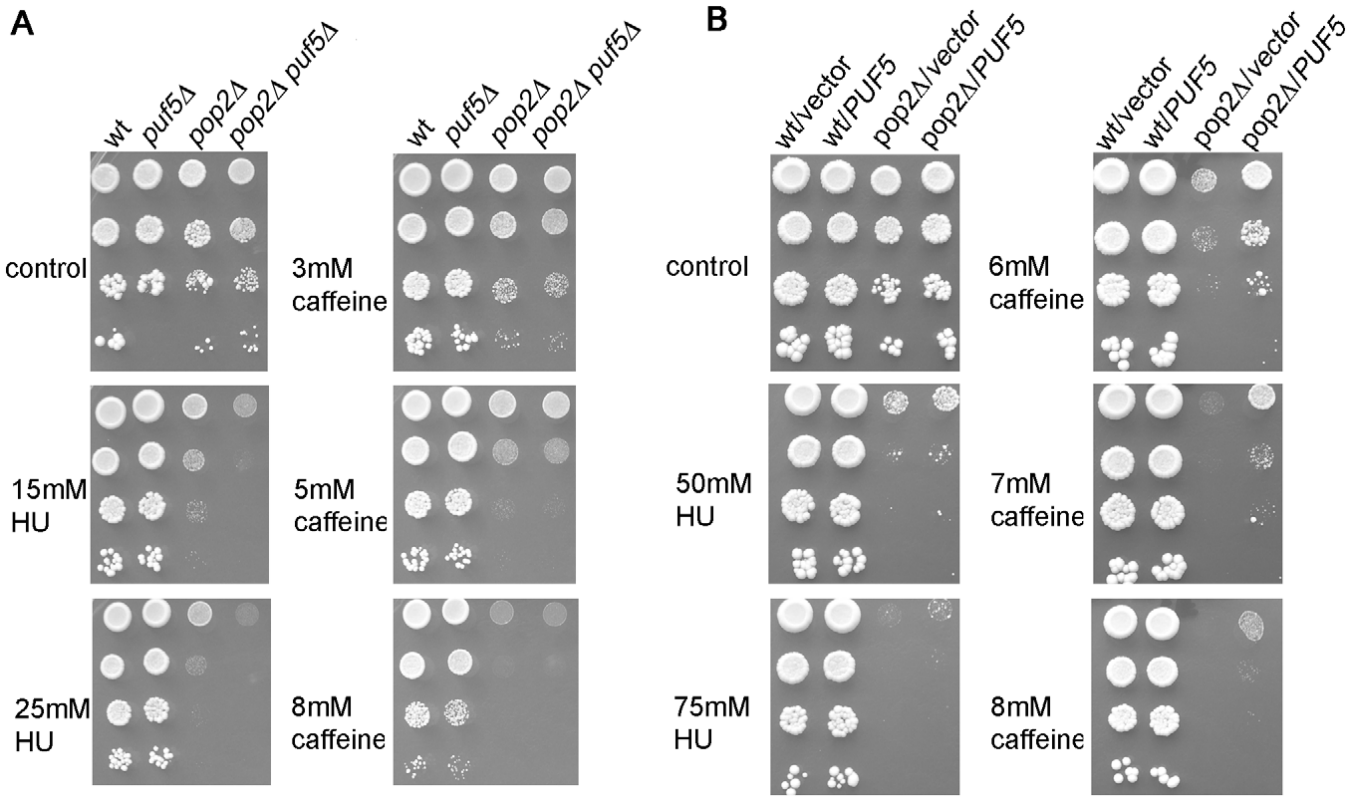
*POP2* and *PUF5* act in the same genetic pathway in response to caffeine. A) Cells of the indicated strains were dropped on plates with or without caffeine and incubated for 2–3 days at 30°C. B) wt or *pop2Δ* mutants (strain YAT179 in Table 1) were transformed with vector only (pRS426) or a 2 µ plasmid expressing Puf5 (pMPT5, 19). Cells were dropped on YPD plates with or without HU and caffeine and the indicated doses and photographed after five days at 30°C. Similar results were obtained on −Ura selective plates (Figure S2).

We therefore tested if double *pop2Δ puf5Δ* mutants also displayed synthetic sensitivity to caffeine (Figure 4A). Unlike what we observed in response to HU, *PUF5* and *POP2* acted in the same genetic pathway in response to caffeine, as the double mutant displayed the same sensitivity across a range of caffeine doses as the more sensitive single *pop2Δ* strain (Figure 4A). This genetic result suggests that, in contrast to what we observed in HU, the functions of Puf5 in response to caffeine are mediated by Pop2-dependent mechanisms.

Puf5 can act as a multi-copy suppressor of some *pop2* mutant phenotypes [9]. We used our findings that *POP2* and *PUF5* act in the same genetic pathway in response to caffeine, but in a different pathway in response to HU, to try to understand the basis of the suppression phenotype. Two possible explanations for the suppression are: (i) overexpression of Puf5 could stabilise protein-protein interactions with other components of the deadenylase complex (which are weak at physiological Puf5 levels), thus enabling Puf5-dependent recruitment of the deadenylase to mRNAs in the absence of Pop2. This explanation predicts that multi-copy Puf5 will suppress the sensitivity of *pop2Δ* mutants to caffeine (because *PUF5* and *POP2* act in the same genetic pathway in response to caffeine, Figure 4A), but not the sensitivity to HU (as Puf5 has Ccr4-Pop2 deadenylase-independent functions in HU, Figure 1); (ii) the suppression of *pop2* phenotypes by multi-copy Puf5 could be resulting from a Pop2-independent role of Puf5, which does not involve recruitment of the deadenylase to transcripts. In that case, we could expect multi-copy Puf5 to suppress both caffeine and HU sensitivity of *pop2Δ*.

We found that multi-copy Puf5 (expressed from a 2 µ plasmid pMPT5) [19] was able to suppress the sensitivity of *pop2Δ* mutants to caffeine, but not to HU (Figure 4B; experiments on YPD plates are shown, but similar results were also obtained on selective–Ura plates, see Figure S2). This result supports the first explanation, ie it indicates that the suppression of *pop2Δ* by multi-copy Puf5 is resulting from Puf5-dependent recruitment of the deadenylase complex to mRNAs, rather than a deadenylase-unrelated function. Previous results that multi copy Puf5 can also suppress the *ccr4* mutant, but not the *dhh1* mutant [9], indicate that increased levels of Puf5 could be bypassing the requirement for Pop2 in recruitment of Dhh1 to mRNA. This predicts that deletion of *DHH1* in a *pop2Δ* background will preclude suppression by multi-copy Puf5, but the experiment could not be performed due to synthetic lethality of *pop2Δ dhh1Δ* mutants [33].

The ability of multi-copy Puf5 to suppress the sensitivity of *pop2Δ* to caffeine, but not to HU, further supports the notion that Puf5 has Pop2-independent roles in response to HU.

**Figure 5:**
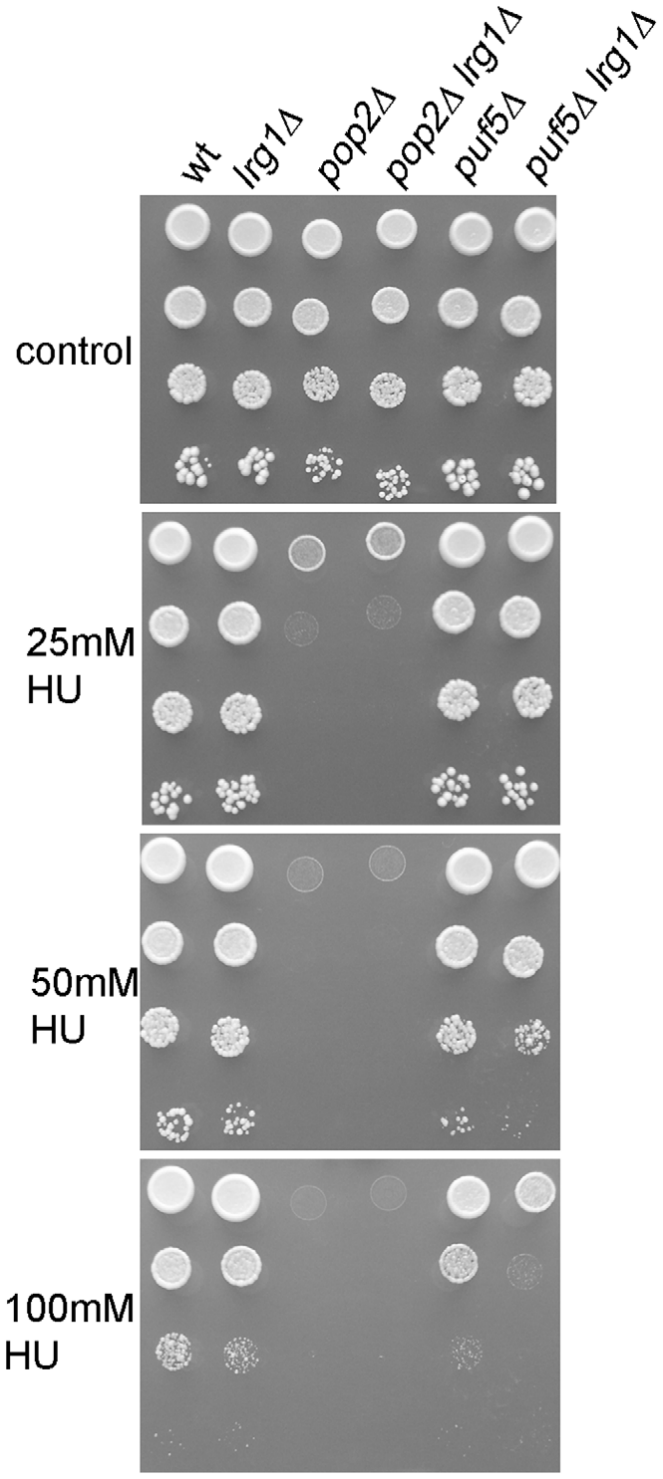
*LRG1* is not the relevant mRNA target for the roles of Puf5 in response to HU. Cells of the indicated strains were dropped on plates with or without HU and photographed after three days of growth at 30°C.

To further address the link between cell wall and HU functions of Puf5, we deleted *LRG1* in the *puf5Δ* and *pop2Δ* mutants and asked if this would suppress their sensitivity to HU (we could not obtain *pop2Δ puf5Δ lrg1Δ* mutants, possibly suggesting either slow growth or synthetic lethality in the triple mutants). Lrg1 is an inhibitor of cell wall biogenesis and cell wall integrity signalling. *LRG1* mRNA levels are kept low in wild type cells by a Puf5-dependent mechanism, and defects in cell wall integrity pathway activation in *puf5Δ* mutants can be partially suppressed by deletion of *LRG1* [19]. In contrast, deletion of *LRG1* resulted in synthetic HU sensitivity when combined with *puf5Δ* and had no effect on HU sensitivity of *pop2Δ* mutants (Figure 5). This result is consistent with the notion that the roles of Puf5 in response to DNA replication and cell wall stress are separate, likely resulting from regulation of a different subset of mRNAs by Pop2-dependent and–independent mechanisms.

### The RNA binding activity of Puf5 is required for resistance to HU

To explain how Puf5 performs roles in HU independently of Pop2, we considered the possibility that it acts in a manner unrelated to gene expression control. For example, Puf3 contributes to mitochondrial morphology by bridging mitochondrial motility factors via protein-protein interactions [18].

This “non-gene expression control” function of Puf5 would presumably not require a functional RNA binding domain. To test this, we mutated two residues required for binding to RNA, S454 and N455, to alanine and asked whether the RNA binding domain mutant can suppress the HU sensitivity phenotype of *puf5Δ*. These experiments were performed using the W303 strain background, in which the *puf5Δ* mutant shows a more pronounced HU sensitivity than in our standard background KY803 (Figure S3). The W303 *puf5Δ* also displayed a more pronounced sensitivity to caffeine, than the same mutant in the KY803 background (Figure S3). This difference in sensitivity of *puf5Δ* is possibly due to the presence of the *ssd1-d2* allele in wild type W303 [34], which has been shown to have synthetic genetic interactions with *puf5Δ* [24].

**Figure 6:**
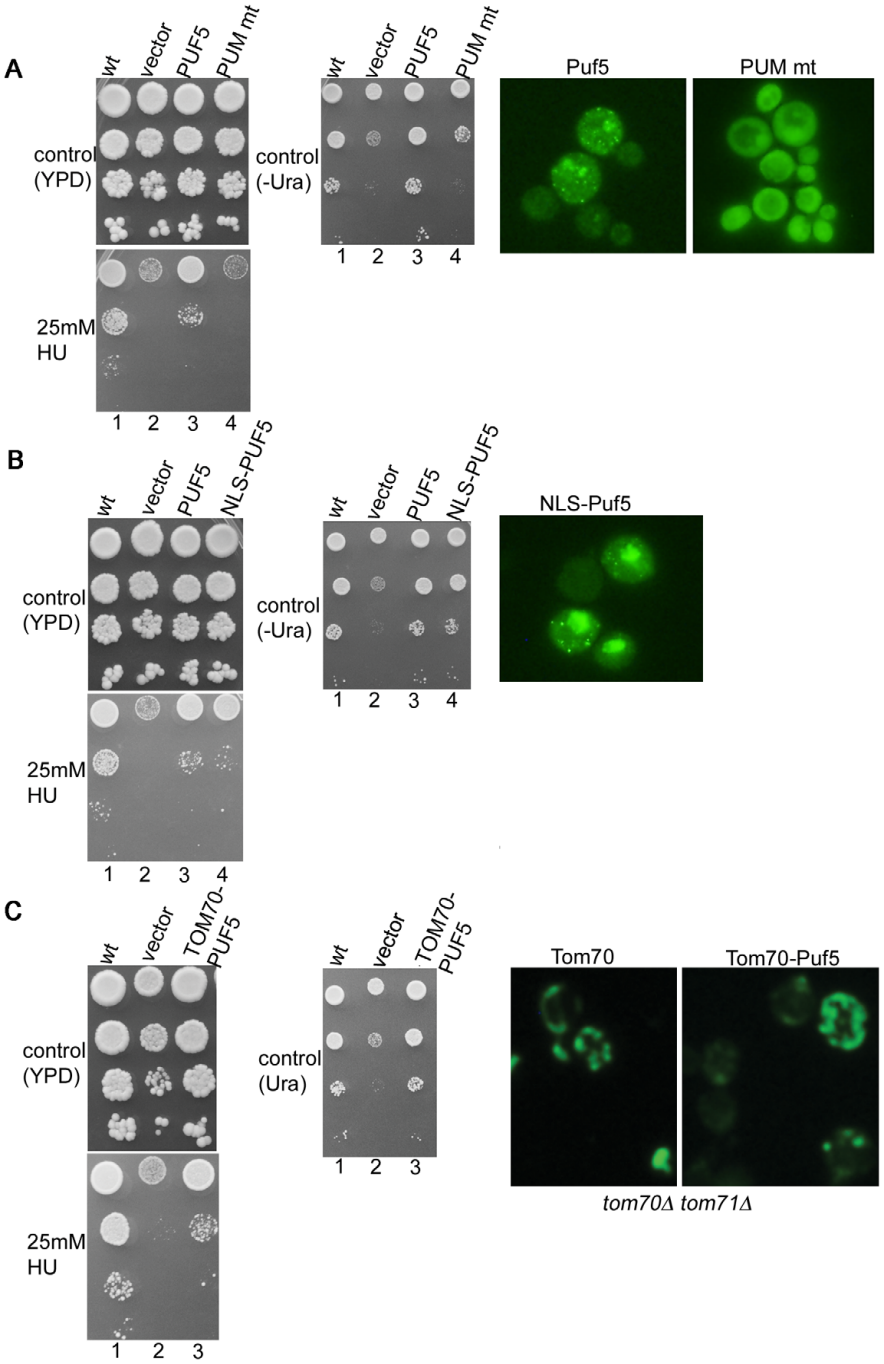
A functional Puf5 RNA binding domain is required and cytoplasmic localization is sufficient for resistance to HU treatment. The experiments were performed in the W3031-A background, in which deletion of *PUF5* alone leads to a strong HU sensitivity phenotype. A) 1-wild type cells transformed with empty vector; 2–4 *puf5Δ* mutants transformed with vector only, or vector expressing wild type Puf5 or the RNA binding domain mutant PUM mt. GFP is fused to the N-terminus of the Puf5 proteins (wild type and PUM mt) for monitoring localization. For survival experiments, cells were grown and spotted on YPD plates ±HU. A −Ura control plate shows that the cells did not lose the plasmid during cell division. For microscopy, cells were grown in −Ura media. Live cells were viewed and photographed. B) The experiments were performed as in A), with wild type Puf5 or an NLS-Puf5 fusion. C) Survival experiments were performed as in A). Micrographs show immunofluorescence experiments to detect Tom70-Puf5 with an anti-Tom70 antibody.

We found that the RNA binding domain mutant (PUM mt) was not able to complement the HU sensitivity of a *puf5Δ* strain (Figure 6A, columns 3 and 4), demonstrating that a functional RNA binding domain of Puf5 is required for growth in HU.

Our Puf5 constructs are GFP fusions, for monitoring protein levels and localisation. We could not detect GFP-Puf5 on Western blots with the anti-GFP antibody, presumably due to low expression levels (not shown). However, microscopy showed that both wild type and PUM mt construct are expressed. As far as we could judge, the expression of the PUM mt mutant was not lower than that of wild type Puf5. Wild type Puf5 localised to cytoplasmic foci in ≈80% of cells (although the intensity of the focal signal varied a lot between cells, Figure 6A). Focal localisation of Puf5 is consistent with a previous report [16]. The PUM mt mutant showed a more uniform cytoplasmic distribution, with foci observed occasionally (Figure 6A and data not shown).

### Puf5 functions in the cytoplasm to promote resistance to HU

Pop2-dependent processes of mRNA deadenylation and translational control are cytoplasmic functions. Perhaps Puf5 has a role in nuclear RNA degradation processes. To explore this possibility, we sent Puf5 to the nucleus by fusing the nuclear localisation signal of SV40 to GFP-Puf5 (NLS-PUF5 in Figure 6B). The NLS-Puf5 fusion protein could complement the HU sensitivity of *puf5Δ* (Figure 6B). However, microscopy showed that NLS-Puf5, even though predominantly nuclear, still localised to cytoplasmic foci in a number of cells (an average of 16.5%±1.8 (standard deviation) of cells had visible Puf5 foci, Figure 6B). It was therefore possible that the cytoplasmic foci are responsible for complementation, rather than the nuclear pool of Puf5.

To distinguish between these possibilities, we restricted the localisation of Puf5 to the cytoplasm, by anchoring Puf5 to the mitochondrial outer membrane with a fusion to the transmembrane domain of the mitochondrial protein Tom70 [35]. In this construct, Puf5 is facing the cytoplasm and can contact its partners [35].

Tom70-Puf5 localised to mitochondria as expected (Figure 6C, right panel). Again, the expression level of the fusion protein was low (in only ≈1% of cells could we observe a signal of similar intensity as the Tom70 vector, Figure 6C). Even so, Tom70-Puf5 could complement the HU sensitivity of *puf5Δ*, demonstrating that restricting Puf5 to the cytoplasm does not impair its function in response to HU. Collectively, the experiments suggest that the pool of Puf5 in cytoplasmic foci is promoting resistance to HU.

## Discussion

Puf5 is a prototypical PUF protein and is the focus of studies to understand the mechanistic details of PUF protein function. Here we used genetic analysis to show that Puf5 has roles in response to DNA replication stress that do not involve Pop2. In contrast, in response to the cell wall integrity pathway activator caffeine, *PUF5* and *POP2* acted in the same genetic pathway, suggesting that the cell wall integrity functions or Puf5 are mediated by Pop2-mediated gene repression mechanisms. In support of a differential requirement for Pop2-mediated mechanisms in gene regulation by Puf5, multi-copy Puf5 could suppress the caffeine sensitivity of *pop2Δ*, but not the sensitivity to HU-induced DNA replication stress. Moreover, deletion of the Puf5 target *LRG1*, which has been reported to suppress defects in cell wall integrity pathway activation [19], resulted in increased sensitivity to HU in a *puf5Δ* background. Taken together, our results are consistent with a model in which Puf5 regulates mRNA expression by Pop2-dependent and Pop2-independent mechanisms. The Puf5 mRNA targets required for cell wall integrity are mainly regulated by Pop2-dependent mechanisms, whereas the mRNA targets required for the response to DNA replication stress are also regulated by Pop2-independent mechanisms.

What is the Pop2-independent mechanism used by Puf5? A functional RNA binding domain of Puf5 was required for growth in HU, and it is thus unlikely that the Pop2-independent role is unrelated to gene expression control. In *Drosophila*, the co-factor Nos interacts with the PUF protein Pum and the Not4 subunit of Ccr4-Pop2-NOT, bypassing Pop2 and bridging Pum to the deadenylase [12]. However, a similar mechanism is unlikely for Puf5: no equivalent of Nos has been identified in yeast and our data shows that Puf5 has functions in response to HU that do not require the Ccr4-Pop2 deadenylase.

In *Xenopus* and *C. elegans* PUF proteins have been implicated in activating gene expression, in combination with translational activation mediated by a cytoplasmic polyadenylation element [36], or by physical interactions with the GLD-2 poly(A) polymerase [13]. In *Saccharomyces cerevisiae*, mRNAs are polyadenylated exclusively in the nucleus and no cytoplasmic polyadenylases have been identified. Our data demonstrates that Puf5 acts in the cytoplasm to promote survival in HU and therefore is unlikely to participate in polyadenylation.

PUFs have also been implicated in mRNA localisation and localised translation. For example, the *C. elegans* PUF FBF-1 might have a role in localised mRNA translation in olfactory neurons [37]. In yeast, Puf3 localises mitochondrial transcripts to mitochondria [38] and Puf6 restricts translation of the *ASH1* mRNA during transport to the bud tip [39]. Puf5 contributes to co-localisation of *PEX14* mRNA with peroxisomes [40]. Our data shows that Puf5 cytoplasmic foci were sufficient for promoting growth in the presence of HU, even when the majority of the protein was sent into the nucleus by a fusion to the SV40 NLS (Figure 6B). We suggest the cytoplasmic rather than the nuclear pool is enabling survival, because restricting the localisation of Puf5 to the cytoplasm (by anchoring to the mitochondrial outer membrane) did not impair survival. Five of the yeast PUFs (Puf1-5) localise to cytoplasmic foci and their function could be to localise functionally related mRNAs for co-regulated translation [16]. We suggest that the Pop2-independent function for Puf5 could involve mRNA localisation for spatial control of translation.

The largest functional group of transcripts bound by Puf5 is those encoding chromatin- modifying functions, such as histone acetylases and deacetylases, methyl-transferases and ATP-dependent chromatin remodelling complexes [16]. Importantly, Puf5 binds to mRNAs encoding several subunits of the SAGA acetyltransferase complex and several subunits of the RSC chromatin remodelling complex [16]. Puf5-mediated co-localisation and co-regulated translation of mRNAs encoding subunits of the same complex might be required for proper complex assembly [16]. Regulation of chromatin structure is important for DNA replication and repair [41]–[42], and therefore improper translation/assembly of chromatin modification complexes in the absence of Puf5 could be affecting the resistance of cells to HU treatment. In support of this notion, deletion of the genes encoding the Sir2–4 complex required for heterochromatin formation suppresses the HU sensitivity of a *puf5Δ* strain [22].

Puf5 has been used as a prototype for understanding the mechanistic aspects of PUF protein function. The only interpretation of the *pop2Δ puf5Δ* mutant's synthetic HU sensitivity is that Puf5 has Pop2-independent functions. This data provides evidence that, in addition to the characterised Pop2-dependent gene repression, Puf5 employs other means to control the expression of its target mRNAs.

## Materials and Methods

### Strains, plasmids and growth conditions

Yeast strains are listed in Table 1. Mutants were constructed by standard methods using PCR and homologous recombination. Growth conditions were YPD (1% yeast extract, 2% peptone, 2% glucose) or selective media lacking uracil at 30°C, 200 rpm.

**Table 1:** Yeast strains.

KY803 strain background		
Strain	Genotype	Reference or Source
YAT9 (wt)	*MATa trp1Δ 1 ura3-52 gcn4 leu2::PET56*	[44]
YAT10	*ccr4Δ::klURA*	[21]
YAT11	*dhh1Δ::NAT*	this study
YAT54	*pop2Δ::klURA*	[21]
YAT61 (*ccr4-1*)	*ccr4-E556A*	[21]
YAT62	*puf4Δ::KAN*	this study
YAT63	*puf5Δ::KAN*	this study
YAT65	*ccr4Δ::klURA puf5Δ::KAN*	this study
YAT66	*ccr4-E556A puf4Δ::KAN*	this study
YAT67	*ccr4-E556A puf5Δ::KAN*	this study
YAT68	*pop2Δ::klURA puf5Δ::KAN*	this study
YAT82	*ccr4Δ::klURA dhh1Δ::NAT*	this study
YAT84	*dhh1Δ::NAT puf5Δ::KAN*	this study
YAT132	*pop2Δ::NAT puf5Δ::KAN*	this study
YAT179	*pop2Δ::LEU2*	Clyde Denis
YAT140	*pan2Δ::KAN*	this study
YAT141	*pan2Δ::KAN ccr4-E556A*	[27]
YAT147	*pan2Δ::KAN puf5Δ::NAT*	this study
YAT149	*ccr4Δ::klUra dhh1Δ::NAT puf5Δ::NAT*	this study
YAT219	*pan2Δ::KAN ccr4-E556A puf5Δ::NAT*	this study
YAT308	*lrg1Δ::NAT*	this study
YAT311	*pop2Δ::klURA lrg1Δ::NAT*	this study
YAT314	*puf5Δ::KAN lrg1Δ::NAT*	this study

W3031-A strain background		
Strain	Genotype	Reference or Source
YAT42 (wt)	*Mata ADE2 in rDNA RAD5+ leu2–3, 112 his3–11, 15 ura3-1 trp1-1 can1–100*	[45]
YAT45	*puf5Δ::NAT*	this study

KlUra - K*luyveromyces lactis URA3*.

Fusions of wild type or PUM mt Puf5 to GFP were constructed by cloning *PUF5* into the GFP-expressing vector p416MET25 HDEL (*CEN/ARS*, *URA3*) [43]. GFP is fused to the N-terminus of Puf5 and the fusion is expressed from the *MET25* promoter.

The RNA binding domain mutant PUM mt was obtained by site-directed mutagenesis of S454 and N455 to alanine [6], using primers F PUM MUT 5′-CTTGTTTGAAGTTCTCCGCCGCTGTTGTGGAAAAATTCATTAAAAAATTATTTAG-3′ and R PUM MUT 5′-CTAAATAATTTTTTAATGAATTTTTCCACAACAGCGGCGGAGAACTTCAAACAAG-3′.

The NLS-Puf5 construct was generated by fusing the SV40 NLS (PKKKRKVE) to the N-terminus of Puf5 and then cloning the NLS-Puf5 fusion into the GFP vector as above.

In Tom70-Puf5, Puf5 is fused to the C-terminus of the transmembrane domain of Tom70 (amino acids 1–98), in plasmid pTOM70 (ΔTPR1–11) [35]. The parental plasmid is p416MET25HDEL.

Multi-copy Puf5 was expressed from plasmid pMPT5 (2 µ,*URA3*) [19] and the vector control was pRS426 (2 µ, *URA3*).

### HU sensitivity assays

HU sensitivity assays were performed as described [21]. 0.2 M HU was added to log phase cultures and samples were collected after 5, 16, 20 and 24 hours. Survival is expressed as percentage of colony formation relative to the 0 time point, which is set to 100%. Averages of at least three independent colonies are shown and the error bar is the standard deviation.

For suppression assays by multi-copy Puf5 (Figure 4B and Figure S2), strains were transformed with either pRS426 (vector control) and pMPT5 [19] and transformants selected on –Ura plates. The assays were done by dropping 10X serial dilutions of cells (starting from OD^600^ = 0.5) onto YPD or –Ura plates with or without HU or caffeine at the doses indicates in the Figures. The strain background was KY803.

For plasmid complementation assays in Figure 6, yeast strains (W3031-A background) were transformed with the relevant plasmids and transformants selected on plates lacking uracil. For HU sensitivity experiments, plasmid-transformed strains were grown in YPD media and spotted on YPD plates with or without HU. We used YPD plates because we could not obtain consistent results with –Ura plates containing HU, possibly due to slow growth rates of *puf5Δ* mutants upon re-growth in selective media. The plasmids are centromeric plasmids unlikely to be lost during cell division; we also included a –Ura control plate in every experiment to confirm that the cells still contain the plasmids.

### Microscopy

For assessing morphology in response to HU treatment, cells (KY803 strain background) were grown to mid log phase and then treated with 0.2 M HU for 16 h. Cells were fixed in 70% ethanol and rehydrated in phosphate buffered saline (PBS) before viewing. A Zeiss Axiovert 25 microscope was used with the 100× magnification objective and photographs were taken on a Kodak film at an original magnification of 250×.

For assaying the localisation of GFP-Puf5, wild type W3031-A cells expressing Puf5, PUM mt or NLS-Puf5 fused to GFP were grown to log phase in -Ura media. Live cells were viewed with an Olympus BX51 microscope. Photographs were taken with the DP Controller software.

To exclude signals from the endogenous Tom70, localization of the Tom70-Puf5 fusion was done in a *tom70Δ tom71Δ* strain [35], by immunofluorescence with an anti-Tom70 antibody. Immunofluorescence was performed as described [29]. Brightness and contrast of the micrographs were adjusted using Photoshop. Foci were counted in at least 140 cells per Puf5 construct. For NLS-Puf5 two independent cultures were counted.

### FACS analysis

0.2 M HU was added to log phase cultures, followed by a 3 h incubation. The asynchronous sample was taken before HU addition (Figure 1B). The 0 time point sample was taken after 3 h in 0.2 M HU, after which cells were spun down, washed and resuspended in YPD without HU for recovery. Recovery was monitored every 15 minutes, for 75 minutes. At each time point, cells were spun down, resuspended in 70% ethanol and stored at 4°C. For FAC analysis, cells were treated with 0.25 mg/ml RNAse A in PBS (phosphate buffer saline) over night at 30°C, stained with 10 µg/ml propidium iodide (30 minutes, room temperature) and sonicated. The analysis was done on a FAC Calibur, using the CellQuest Pro software.

## Supporting Information

Figure S1 *POP2* and *PUF5* act in separate genetic pathways in response to HU A) Two independent *pop2Δ puf5Δ* double mutants (YAT68 and YAT132) were tested for synthetic hypersensitivity to HU. 10X serial dilutions starting from OD600 = 0.5 were dropped on plates with or without HU and photographed after 3 days at 30°C. B) pMPT5 or empty vector pRS426 were transformed into *pop2Δ puf5Δ* double mutants (YAT132 in Table 1), to test whether plasmid borne *PUF5* can complement the synthetic sensitivity to HU. Cells were grown on -Ura plates with or without HU for five days and photographed.
(0.29 MB TIF)

Figure S2 Suppression of *pop2Δ* phenotypes by multi-copy Puf5 Wild type (wt) or *pop2Δ* mutants were transformed with pMPT5 (2 µ, *URA3*) or pRS426 vector only control (2 µ, *URA3*) and dropped on -Ura plates with or without caffeine and HU. Cells were photographed after five days of growth at 30°C.
(0.30 MB TIF)

Figure S3 The phenotypes of *puf5Δ* are more pronounced in the W3031-A strain background Cells of wild type and *puf5Δ* mutants in the W3031-A background were dropped on caffeine or HU containing plates and photographed after three days of growth.
(0.31 MB TIF)
