# Supplementary figures and images for: The Yeast PUF Protein Puf5 Has Pop2-Independent Roles in Response to DNA Replication Stress
Source: PLoS One. 2010 May 14;5(5):e10651. doi: 10.1371/journal.pone.0010651 (PMC2871046; doi:10.1371/journal.pone.0010651)

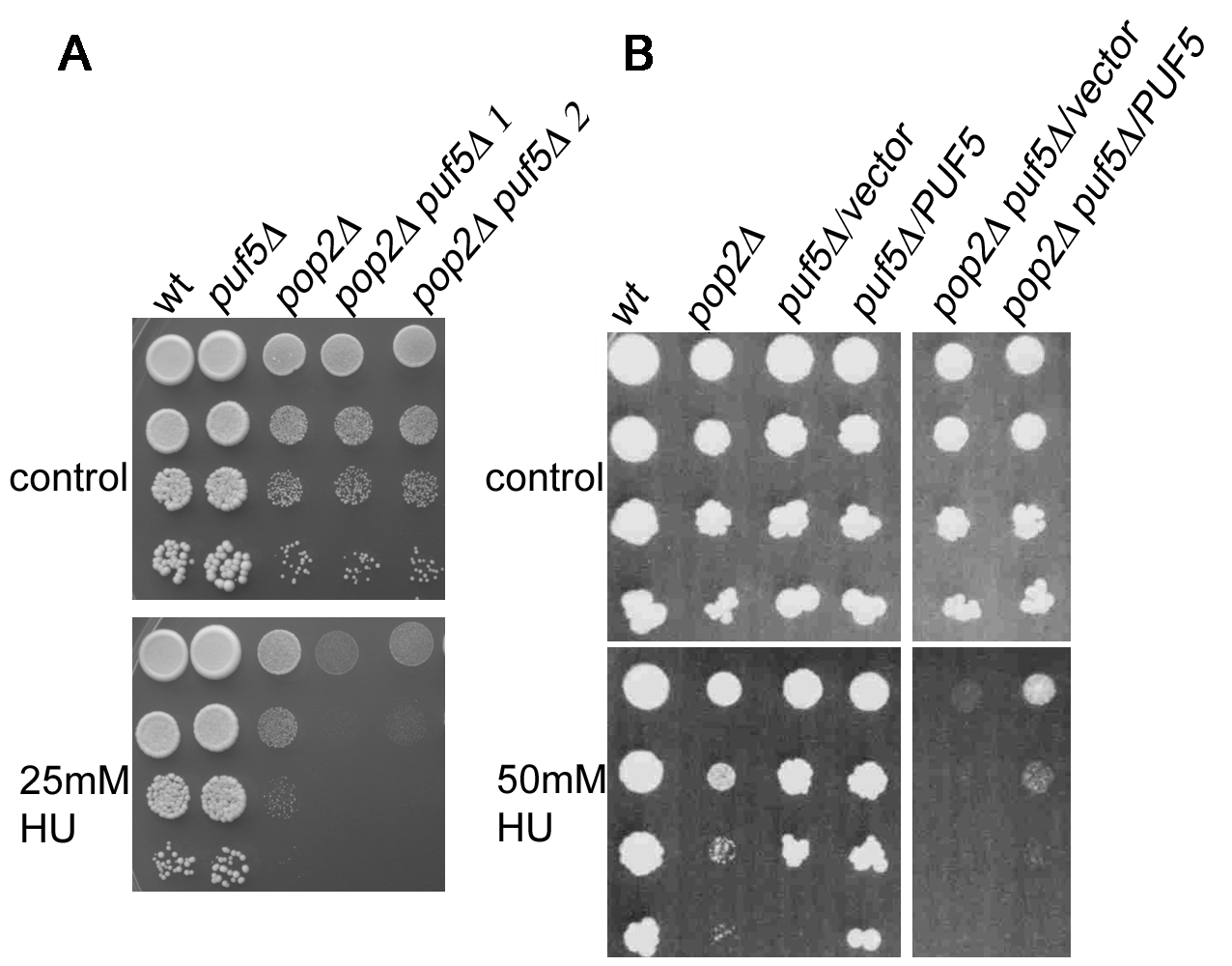

Supplement: Figure S1 — POP2 and PUF5 act in separate genetic pathways in response to HU A) Two independent pop2Δ puf5Δ double mutants (YAT68 and YAT132) were tested for synthetic hypersensitivity to HU. 10X serial dilutions starting from OD600 = 0.5 were dropped on plates with or without HU and photographed after 3 days at 30°C. B) pMPT5 or empty vector pRS426 were transformed into pop2Δ puf5Δ double mutants (YAT132 in Table 1), to test whether plasmid borne PUF5 can complement the synthetic sensitivity to HU. Cells were grown on -Ura plates with or without HU for five days and photographed. (0.29 MB TIF) [file pone.0010651.s001.tif]

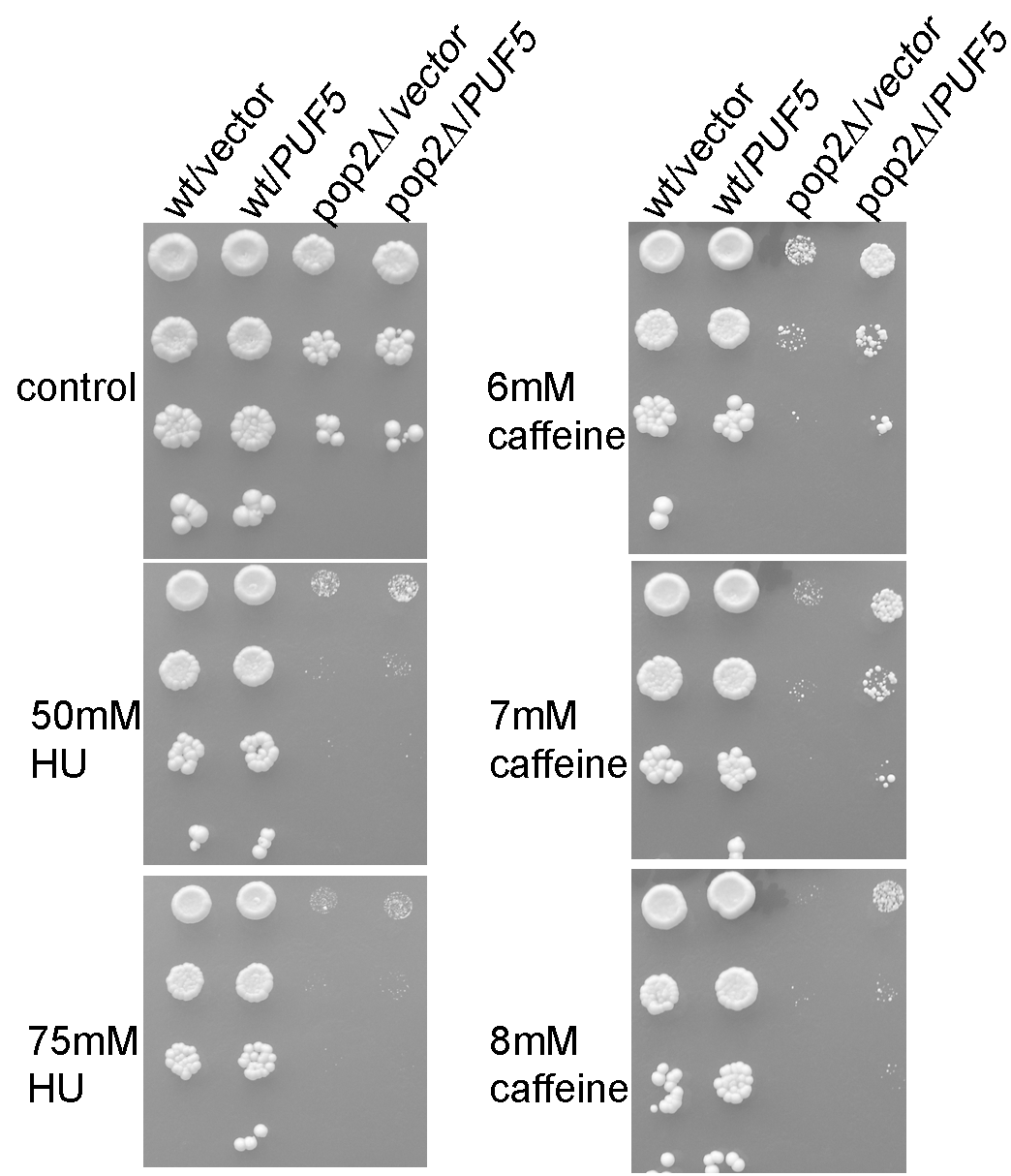

Supplement: Figure S2 — Suppression of pop2Δ phenotypes by multi-copy Puf5 Wild type (wt) or pop2Δ mutants were transformed with pMPT5 (2 µ, URA3) or pRS426 vector only control (2 µ, URA3) and dropped on -Ura plates with or without caffeine and HU. Cells were photographed after five days of growth at 30°C. (0.30 MB TIF) [file pone.0010651.s002.tif]

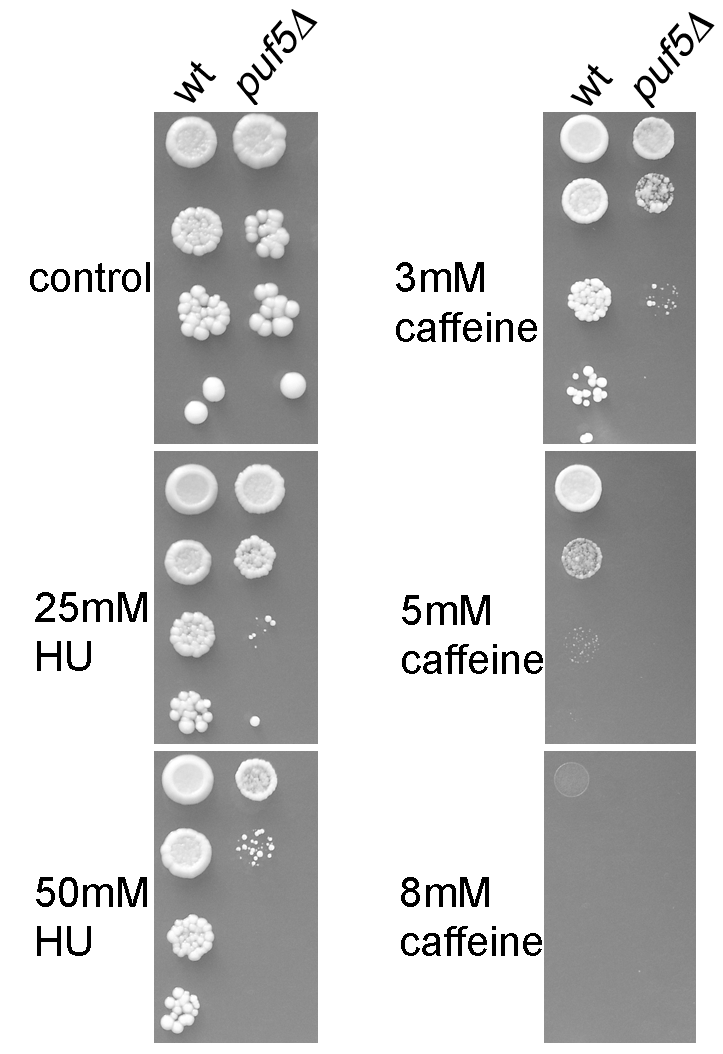

Supplement: Figure S3 — The phenotypes of puf5Δ are more pronounced in the W3031-A strain background Cells of wild type and puf5Δ mutants in the W3031-A background were dropped on caffeine or HU containing plates and photographed after three days of growth. (0.31 MB TIF) [file pone.0010651.s003.tif]
